# The application value of systematic nursing in severe acute pancreatitis patients undergoing blood purification treatment

**DOI:** 10.1097/MD.0000000000042013

**Published:** 2025-05-02

**Authors:** Lan Fang, Mengmeng Wang, Xiaoling Ye, Jiehua Wu, Liang He

**Affiliations:** a Department of Emergency ICU, The Second Affiliated Hospital, Jiangxi Medical College, Nanchang University, Nanchang, China; b Department of Emergency, The Second Affiliated Hospital, Jiangxi Medical College, Nanchang University, Nanchang, China.

**Keywords:** continuous renal replacement therapy, inflammatory response, nursing satisfaction, severe acute pancreatitis, systematic nursing

## Abstract

This study aimed to assess the effects of systematic nursing on severe acute pancreatitis (SAP) patients undergoing continuous renal replacement therapy (CRRT). In this retrospective cohort study, data from SAP patients who received CRRT in our hospital’s ICU from January 2022 to January 2024 were analyzed. Patients were grouped based on nursing care type: the experimental group (systematic nursing, 41 cases) and the control group (routine nursing, 59 cases). Observed indicators included vital sign stability, pain and anxiety scores, serum biochemical markers (serum amylase, C-reactive protein [CRP], and white blood cell count), complication rates, and nursing satisfaction. Propensity score matching was used to control for baseline differences. The experimental group demonstrated superior clinical outcomes compared to the control group, with significantly more stable body temperature (36.76 ± 0.31°C vs 37.05 ± 0.45°C, *P* = .036) and heart rate (82.1 ± 5.7 bpm vs 84.9 ± 6.6 bpm, *P* = .046). Pain (3.2 ± 1.1 vs 4.5 ± 1.3, *P* = .012) and anxiety scores (2.8 ± 0.9 vs 3.6 ± 1.0, *P* = .021) were also significantly lower. Inflammatory markers such as serum amylase (95.4 ± 30.2 vs 120.5 ± 35.8, *P* = .004), CRP (7.2 ± 3.1 vs 10.3 ± 3.5, *P* = .005), and white blood cell count (8.5 ± 2.0 vs 10.1 ± 2.6, *P* = .007) decreased significantly in the experimental group. Furthermore, the experimental group had lower complication rates than the control group (MODS incidence: 23.53% vs 73.53%, *P* = .030; infection rate: 29.41% vs 88.24%, *P* = .014; renal insufficiency incidence: 14.71% vs 58.82%, *P* = .026) and higher nursing satisfaction (*P* = .041). Systematic nursing significantly improved clinical outcomes in SAP patients receiving CRRT, enhancing vital sign stability, reducing pain and anxiety, controlling inflammation, decreasing complication rates, and increasing nursing satisfaction. These results support the use of systematic, individualized nursing in managing critically ill SAP patients.

## 1. Introduction

Severe acute pancreatitis (SAP) is a common critical condition that develops rapidly, often arising from acute pancreatitis (AP). Globally, the incidence of AP is 13 to 45 cases per 100,000 people annually. Over the past 20 years, the incidence of AP in China has risen from 0.19% to 0.71%, with approximately 20% of cases progressing to moderate or SAP.^[1–4]^ Studies indicate that SAP patients may experience persistent organ failure early in the disease, with a mortality rate reaching 67% within the first week. The primary mechanism of organ failure in SAP patients is systemic inflammatory response syndrome (SIRS), which leads to the accumulation of inflammatory mediators and metabolic toxins (e.g., IL-6 and C-reactive protein). Continuous blood purification, also known as continuous renal replacement therapy (CRRT), is widely used to remove these inflammatory mediators, interrupt SIRS, and prevent further organ damage, serving as an essential treatment for SAP internationally.^[5–8]^

The effectiveness of CRRT in removing inflammatory mediators largely depends on the adequacy of the treatment. However, due to operational challenges, frequent unplanned interruptions occur during CRRT, significantly reducing treatment efficacy. Studies show that these interruptions are often caused by factors such as insufficient anticoagulation, frequent machine alarms, catheter-related infections, and patient noncooperation, which diminish treatment effectiveness and increase patient burden. Previous studies have explored various nursing interventions. Researchers have found that sufficient preparation, increased blood flow rate, effective analgesia, and regular saline flushing can help prevent clotting and unplanned interruptions, thereby enhancing CRRT efficacy. Currently, there is a lack of evidence-based guidelines on clinical interventions to reduce CRRT interruptions, which hampers quality management and process monitoring in nursing care.

Systematic nursing is an evidence-based nursing intervention that integrates various evidence-based nursing measures to systematically improve patient outcomes. Therefore, it is necessary to develop evidence-based nursing interventions to ensure the adequacy of CRRT treatment in emergency SAP patients.^[9–11]^ Our research hypothesizes that systematic nursing intervention will improve treatment outcomes, including enhanced stability of vital signs, reduced pain and anxiety, reduced inflammatory response, lower complication rates, and improved nursing satisfaction. Given the absence of studies applying systematic nursing to SAP patients receiving CRRT, this study aims to develop a systematic nursing intervention for CRRT treatment in emergency SAP patients and to evaluate its impact on CRRT outcomes in these patients.

## 2. Materials and methods

### 2.1. Study design

This study was approved by The Ethics Committee of The Second Affiliated Hospital of Nanchang University. This study is a retrospective cohort study that collected case data from SAP patients previously admitted to the emergency ICU in our hospital from January 2022 to January 2024 and who underwent blood purification treatment. Patients were grouped according to the type of nursing care previously received: the experimental group (41 cases) received systematic nursing, while the control group (59 cases) received routine nursing.

*Inclusion criteria*: SAP patients aged ≥ 18 years who required blood purification treatment with a cumulative duration of at least 48 consecutive hours. Complete clinical data were available, including hospitalization records, nursing notes, and detailed records of blood purification treatment. Patients voluntarily agreed to participate and met the ethical committee’s requirements.

*Exclusion criteria*: Patients with other major organ failures (e.g., end-stage renal disease or heart failure), those with severe immune deficiency or uncontrolled infection, patients with psychiatric or cognitive disorders unable to comply with the nursing protocol, and cases with incomplete medical records that precluded accurate evaluation of nursing outcomes.

### 2.2. Treatment details

In this study, SAP patients underwent CRRT with low-dose heparin anticoagulation. Flow rates were adjusted according to patient body weight to 35 to 55 mL/(h·kg), and blood flow rates were maintained at 160 to 240 mL/min to ensure the effective clearance of metabolic waste. The average duration of a single CRRT session was 13.2 ± 6.1 hours, with a total continuous treatment duration averaging 8.1 ± 2.3 days. Treatment frequency and ultrafiltration volume were adjusted as needed based on patient condition to maintain fluid balance and improve metabolic status.

### 2.3. Nursing methods

#### 2.3.1. Control group nursing method: routine nursing

The control group received traditional routine nursing care, including the following:

##### 2.3.1.1. Basic condition information

Nurses provided patients and families with basic information about the disease, including the pathophysiology, progression, and prognosis of SAP, to help them understand the treatment process and its importance.

##### 2.3.1.2. Routine medication management

Following physician orders, nurses managed patients’ medications, ensuring the correct dosage, timing, and method for antibiotics, analgesics, antispasmodics, and other necessary drugs. Prior to administration, allergies were checked, and reactions were monitored and documented for timely feedback.

##### 2.3.1.3. Emergency care

For patients with acute changes in condition, nurses implemented necessary emergency interventions, including maintaining airway patency and managing cardiopulmonary resuscitation equipment, to address potential emergencies promptly.

##### 2.3.1.4. Monitoring

Routine monitoring included vital signs such as temperature, pulse, respiration, blood pressure, and urine output, with regular recording to observe disease progression. Nurses reported abnormalities (e.g., high fever, low blood pressure) to physicians for prompt intervention. In addition, specialized abdominal and Grey-Turner sign assessments were conducted to monitor disease development comprehensively. During blood purification, parameters were adjusted following medical orders, with close attention to machine alarms, including flow, pressure, and filter status, to ensure continuous and safe blood purification. In case of alarms, nurses checked and resolved issues promptly to maintain safe operation.

##### 2.3.1.5. Catheter care

The puncture site was regularly inspected to ensure skin cleanliness and integrity. Dressings were replaced as needed to prevent infection, and catheters were refixed if necessary to avoid treatment interruption due to displacement.

##### 2.3.1.6. Intake and output recording

Daily detailed records of fluid intake and output, especially urine, bowel movements, and infusions, were maintained to ensure fluid balance.

#### 2.3.2. Experimental group nursing method: systematic nursing

In addition to routine care, the experimental group received comprehensive systematic nursing interventions, including:

##### 2.3.2.1. Admission nursing assessment

Upon admission, a detailed assessment was conducted, collecting the patient’s medical history, current condition, and medication records to support the development of a personalized care plan and to anticipate possible complications and nursing challenges.

##### 2.3.2.2. Equipment management

During blood purification treatment, equipment management aimed to ensure the continuous and stable operation of the blood purification system, enhancing treatment efficacy. Specific measures included: Catheter and Connection Sealing Check: Every 2 hours, the integrity of catheter connections was inspected to prevent leaks or air entry, which could compromise treatment. Key Pressure Parameters Monitoring: Filtration pressure, inlet pressure, outlet pressure, and transmembrane pressure were monitored hourly. These parameters were critical for assessing filter permeability and equipment status. Significant changes in transmembrane pressure might indicate impending clotting, requiring preemptive intervention. Alarm Management: Nurses responded promptly to equipment alarms, checking components as indicated and taking corrective measures (e.g., checking catheter patency, clearing air bubbles, readjusting flow rate). Alarms were set per medical orders, and frequent alarms were monitored to identify equipment malfunction or improper blood flow. Filter Status and Replacement: Filters were inspected regularly for clotting or abnormal flow rates. Filters were replaced promptly upon resistance increase or alarm prompts, preventing blood flow blockage and improving purification efficiency.

##### 2.3.2.3. Fluid balance maintenance

In blood purification treatment, fluid balance management is critical to maintaining the patient’s water and electrolyte balance and ensuring treatment efficacy. Specific measures include: Fluid Intake and Output Recording and Assessment: Daily detailed records of the patient’s fluid intake and output, including infusion volume, urine output, drainage, sweating, and other fluid losses, were kept. The fluid balance status was updated every 2 hours, and intake and output volumes were dynamically adjusted based on the patient’s fluid needs to avoid complications from inadequate or excessive fluid load. Ultrafiltration Adjustment: Ultrafiltration volume of the blood purification device was adjusted in real-time based on the patient’s fluid balance and clinical presentation. If the patient showed signs of fluid retention (e.g., edema or difficulty breathing), ultrafiltration volume was increased; in cases of low blood pressure or dehydration risk, ultrafiltration volume was decreased to maintain safe fluid balance.

Coagulation Monitoring: Coagulation function, including prothrombin time and activated partial thromboplastin time, was monitored every 4–6 hours to assess anticoagulation effectiveness and coagulation abnormalities. If abnormal coagulation was detected, the heparin dose was adjusted promptly to avoid filter clotting and patient bleeding. Electrolyte and Biochemical Monitoring: Every 8 hours, electrolyte (e.g., sodium, potassium, calcium, magnesium) and biochemical (e.g., creatinine, blood urea nitrogen) levels were measured to monitor the patient’s metabolic status, ensuring the safety and efficacy of CRRT. If electrolyte imbalances were identified, intravenous supplementation or adjustment of the dialysis solution was used for correction. Clinical Observations: The patient’s weight, skin elasticity, blood pressure, heart rate, and other indicators were closely observed to assess fluid balance effectiveness. If the patient experienced weight gain, skin edema, or increased heart rate, the treatment plan was adjusted promptly to prevent adverse effects from fluid overload or imbalance.

##### 2.3.2.4. Psychological nursing

Through active communication, a trusting relationship was established with the patient and family. The patient was informed about the disease, treatment plan, and precautions, with explanations about the disease’s controllability to help them understand and cooperate with the treatment, reducing anxiety and tension and improving treatment adherence.

##### 2.3.2.5. Infection control management

Strict aseptic protocols were followed, including wearing sterile gloves and practicing hand hygiene during blood purification procedures, catheter care, and dressing changes. The patient’s room was disinfected daily with ultraviolet light to maintain a clean environment, and the oral and nasal cavities, as well as other susceptible areas, were cleaned regularly, especially during fasting or gastrointestinal decompression.

##### 2.3.2.6. Nutritional support

Total parenteral nutrition was provided through a central venous line, tailored to meet the patient’s metabolic needs with nutrition bags rich in protein and essential trace elements. Sterile techniques were rigorously followed to ensure safe nutrition intake. The patient’s nutritional status was monitored closely, and dietitians were consulted as needed to adjust the nutritional plan to meet recovery needs in SAP.

##### 2.3.2.7. Complication prevention and care

For potential complications during blood purification treatment, such as hypotension, bleeding, and filter clotting, nurses provided close monitoring and prompt intervention. If clotting signs appeared in the filter, it was replaced in a timely manner, usually every 6–10 hours. The catheter was securely fixed to prevent displacement, and the puncture site was checked for any signs of bleeding or infection.

##### 2.3.2.8. Follow-up and support

Before discharge, a detailed discharge care plan and health education guide were provided, including dietary recommendations, frequency of follow-up visits, and medication instructions. Patients and families were instructed on monitoring vital signs and recognizing symptoms of complications at home, with contact information provided for consultation and guidance as needed.

### 2.4. Data collection

#### 2.4.1. General indicators

The study’s observational indicators included demographic and baseline characteristics, such as age, gender ratio, BMI, APACHE II score, SOFA score, ICU stay duration, blood purification treatment time, history of hypertension, coronary heart disease, diabetes, smoking, and alcohol use, as well as serum electrolytes (sodium, potassium) and renal function markers (creatinine, blood urea nitrogen). Additionally, observations covered the stability of vital signs (temperature, systolic and diastolic blood pressure, heart rate), pain and anxiety scores, serum biochemical indicators (serum amylase, CRP, white blood cell count), incidence of complications (including multiple organ dysfunction syndrome [MODS], infections, renal dysfunction, bleeding, hypotension, hypothermia, catheter blockage, catheter-related bloodstream infection, electrolyte imbalance, and circulatory overload), and nursing satisfaction (rated as highly satisfied, satisfied, neutral, dissatisfied, or very dissatisfied).^[12,13]^ In this study, we observed that patients frequently developed complications such as MODS and infections. To ensure the comparability of data across different hospitals, we adopted standardized diagnostic criteria for these complications. MODS was defined as the dysfunction of 2 or more organ systems based on established scoring systems (e.g., SOFA score), while infections were diagnosed according to the criteria outlined by the Centers for Disease Control and Prevention and confirmed through microbiological or clinical evidence. These standardized criteria were consistently applied throughout the study to minimize variability in diagnose.

#### 2.4.2. Scoring indicators

(1) **APACHE II Score (Acute Physiology and Chronic Health Evaluation II):**

Used to assess the severity of the patient’s condition and predict mortality risk. This scoring system calculates a composite score based on various physiological and laboratory parameters, including age, temperature, mean arterial pressure, heart rate, respiratory rate, blood oxygen partial pressure, serum electrolytes, blood urea nitrogen, creatinine, hemoglobin, white blood cell count, and Glasgow Coma Scale score. A higher score indicates greater severity and poorer prognosis.^[14]^

(2) **SOFA Score (Sequential Organ Failure Assessment):**

Used to evaluate and monitor multiple organ dysfunction, particularly in ICU patients. The SOFA score includes indicators in 6 areas: respiratory, coagulation, liver, cardiovascular, renal, and central nervous system functions. Each indicator is scored based on severity, with a higher total score indicating a more severe degree of organ failure and a poorer prognosis.

(3) **Pain Score:**

The Numeric Rating Scale was used, with patients rating their pain on a scale of 0 to 10, where 0 represents no pain and 10 represents the most severe pain. This simple method aids in assessing the intensity of the patient’s pain and the effectiveness of interventions.

(4) **Anxiety Score:**

The Self-Rating Anxiety Scale was used, where patients complete a questionnaire reflecting their anxiety levels. Scores range from 40 to 80, with higher scores indicating more severe anxiety.

(5) **Nursing Satisfaction:**

In this study, nursing satisfaction was evaluated by patient self-assessment, with a total score range of 0 to 100, where higher scores indicate greater satisfaction. The rating criteria include aspects such as service attitude, professional skills, communication ability, clarity of treatment explanation, and response speed. Nursing satisfaction was categorized into 5 levels: 90 to 100 points: “Highly Satisfied”—indicating the patient was extremely satisfied with the nursing care. 75 to 89 points: “Satisfied”—indicating overall satisfaction with minor suggestions for improvement. 60 to 74 points: “Neutral”—indicating a generally satisfactory experience with both positive and negative aspects. 45–59 points: “Dissatisfied”—indicating several areas in need of improvement. Below 45 points: “Very Dissatisfied”—indicating significant dissatisfaction with nursing care.

### 2.5. Statistical analysis

The statistical analysis in this study employed multiple methods to comprehensively evaluate the effects of systematic nursing on SAP patients. First, descriptive statistics were used to calculate the means, standard deviations, and frequency distributions of baseline characteristics such as age, gender ratio, BMI, APACHE II score, and SOFA score, to describe the basic profiles of the experimental and control groups. To compare categorical variables (e.g., incidence of complications, nursing satisfaction levels), the Chi-square test was used, while for continuous variables (e.g., temperature, heart rate, pain, and anxiety scores), the independent sample *t* test was applied to determine significant differences between the 2 groups.

To control for baseline differences, propensity score matching (PSM) was used to balance variables such as age, gender, and BMI between the experimental and control groups, and significance tests of baseline characteristics were conducted before and after matching. For changes in variables from baseline to posttreatment (e.g., pain and anxiety scores, serum biochemical markers), paired sample *t* tests were employed to assess the effectiveness of systematic nursing. Through the combined application of these statistical methods, this study evaluated the impact of systematic nursing on reducing complications, stabilizing vital signs, improving psychological well-being, and enhancing nursing satisfaction while minimizing confounding effects.

## 3. Results

### 3.1. Baseline characteristics analysis after propensity score matching

In this study, to control for the influence of confounding factors, we performed PSM for patients in the experimental group (systematic nursing) and the control group (routine nursing) and compared the differences in baseline characteristics before and after matching. Prior to matching, there were significant differences between the 2 groups in variables such as age, gender, BMI, APACHE II score, ICU stay duration, blood purification duration, and history of hypertension and coronary heart disease (*P* < .05), while there were no significant differences in SOFA score, antibiotic use, diabetes, smoking, alcohol consumption, serum creatinine, blood urea nitrogen, sodium, and potassium levels (*P* > .05).

After matching, there were no statistically significant differences in any baseline characteristics (*P* > .05). For example, the average age in the experimental and control groups after matching was 66.5 ± 7.3 years and 66.7 ± 7.6 years, respectively (*P* = .912); the male-to-female ratio was 19/15 and 20/14 (*P* = .854); and the BMI was 25.2 ± 3.2 and 25.4 ± 3.3 (*P* = .881). Other variables, including APACHE II score, SOFA score, ICU stay duration, CRRT duration, antibiotic use, hypertension, diabetes, coronary heart disease, smoking, and alcohol consumption, as well as serum electrolytes and renal function indicators, also showed no significant differences after matching.

Successful PSM eliminated significant baseline differences between the 2 groups, indicating that when comparing the effects of nursing interventions, the inter-group differences were primarily due to nursing methods, with the impact of confounding factors substantially reduced (Table 1).

**Table 1:** Baseline characteristics of patients in experimental and control groups before and after propensity score matching.

Factor	Pre-matching experimental group (n = 41)	Pre-matching control group (n = 59)	z/χ²	*P*-value	Post-matching experimental group (n = 34)	Post-matching control group (n = 34)	z/χ²	*P*-value
Age (years)	68.3 ± 7.8※	63.2 ± 8.5※	2.332	**.021**	66.5 ± 7.3	66.7 ± 7.6	0.184	.912
Gender (male/female)	28/13※	25/34※	7.823	**.005**	19/15	20/14	0.034	.854
BMI (kg/m²)	26.2 ± 3.5※	24.1 ± 3.1※	2.507	**.013**	25.2 ± 3.2	25.4 ± 3.3	0.103	.881
APACHE II score	14.5 ± 3.8※	12.2 ± 3.9※	2.703	**.008**	13.8 ± 3.6	13.9 ± 3.7	0.024	.942
SOFA score	7.6 ± 2.1※	6.8 ± 2.0※	1.546	**.129**	7.0 ± 2.1	7.1 ± 2.0	0.033	.942
Antibiotic use (cases)	36	45	3.907	.051	27	28	0.029	.902
ICU stay duration (days)	12.1 ± 4.5※	10.2 ± 4.3※	2.098	**.039**	11.0 ± 4.1	11.2 ± 4.0	0.041	.91
CRRT duration (days)	8.2 ± 3.0※	7.3 ± 3.1※	1.376	**.049**	8.1 ± 3.2	8.0 ± 3.0	0.029	.977
Hypertension (cases)	20※	12※	4.307	**.038**	15	14	0.073	.787
Diabetes (cases)	12	17	1.328	.249	10	11	0.058	.81
Coronary heart disease (cases)	9※	15※	0.807	**.049**	7	6	0.074	.785
Smoking (cases)	14	20	0.337	.562	10	11	0.058	.81
Drinking (cases)	13	16	0.15	.699	9	10	0.07	.791
Creatinine (mg/dL)	1.24 ± 0.3	1.46 ± 0.4	1.234	.218	1.43 ± 0.31	1.39 ± 0.29	0.052	.823
Blood urea nitrogen (mg/dL)	18.56 ± 3.3※	20.46 ± 2.6※	1.678	**.044**	19.25 ± 3.6	19.54 ± 5.1	0.014	.923
Sodium (mmol/L)	140.26 ± 3.1	139.15 ± 4.7	0.967	.334	139.25 ± 4.3	139.01 ± 3.8	0.029	.978
Potassium (mmol/L)	4.0 ± 0.5	4.2 ± 0.4	0.743	.458	3.9 ± 0.3	4.1 ± 0.4	0.023	.819

CRRT = continuous renal replacement therapy.

### 3.2. Stability of vital signs at baseline and posttreatment in the experimental and control groups

**Figure 1.**
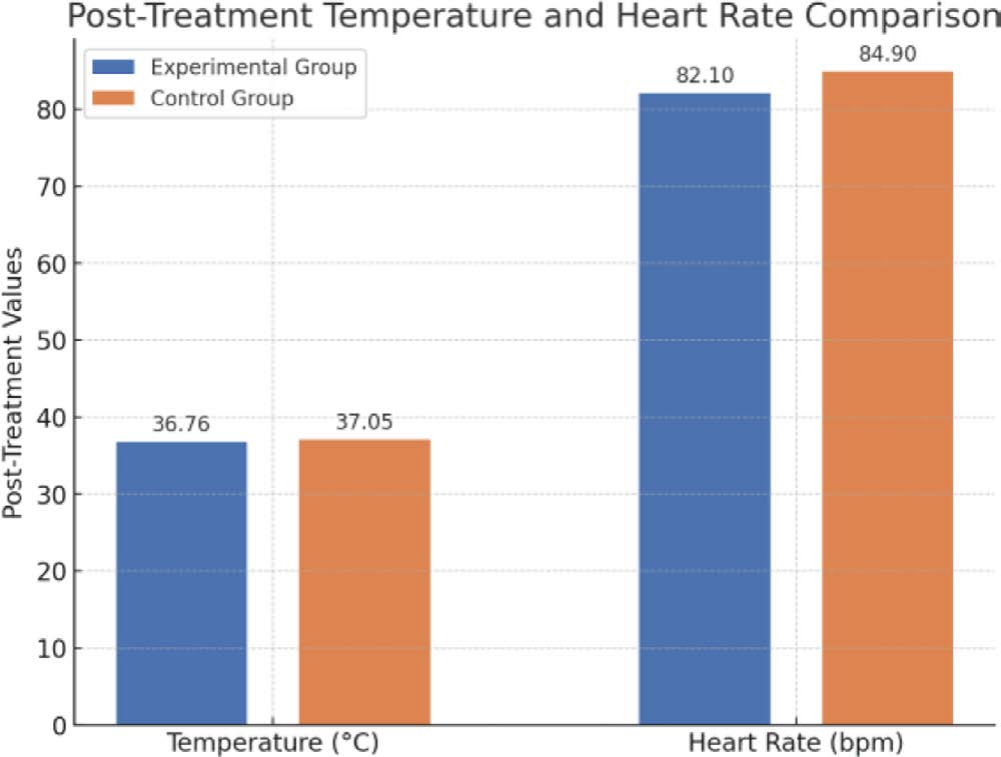
Comparison of posttreatment temperature and heart rate between experimental and control groups with standard deviation.

At baseline, there were no significant differences in vital signs between the experimental and control groups, including temperature, systolic blood pressure, diastolic blood pressure, and heart rate. The baseline temperature in the experimental group was 37.12 ± 0.38 °C, while in the control group it was 37.23 ± 0.49 °C (*P* = .384). The baseline systolic blood pressure in the experimental group was 134.7 ± 11.6 mm Hg, compared to 135.9 ± 12.8 mm Hg in the control group (*P* = .722). Similarly, baseline values for diastolic blood pressure and heart rate were comparable between groups, showing no statistically significant differences (Fig. 1).

Posttreatment, vital signs were more stable in the experimental group than in the control group. The posttreatment temperature in the experimental group (36.76 ± 0.31 °C) was significantly lower than that in the control group (37.05 ± 0.45 °C, *P* = .036). The heart rate in the experimental group was 82.1 ± 5.7 bpm, notably lower than in the control group, which was 84.9 ± 6.6 bpm (*P* = .046). The systolic blood pressure in the experimental group (125.3 ± 10.4 mm Hg) showed a downward trend compared to the control group (130.2 ± 12.3 mm Hg), though this difference approached but did not reach statistical significance (*P* = .055). There was no statistically significant difference in diastolic blood pressure between the 2 groups posttreatment (*P* = .756) (Table 2).

**Table 2:** Baseline and posttreatment stability of vital signs between experimental and control groups.

Vital sign	Experimental group mean ± SD	Control group mean ± SD	*t* value	*P*-value
Temperature baseline (°C)	37.12 ± 0.38	37.23 ± 0.49	0.87	.384
Temperature posttreatment (°C)	36.76 ± 0.31*	37.05 ± 0.45*	2.13	**.036**
Systolic blood pressure baseline (mm Hg)	134.7 ± 11.6	135.9 ± 12.8	0.36	.722
Systolic blood pressure posttreatment (mm Hg)	125.3 ± 10.4*	130.2 ± 12.3*	1.93	.055
Diastolic blood pressure baseline (mm Hg)	85.2 ± 8.7	85.8 ± 9.4	0.31	.756
Diastolic blood pressure posttreatment (mm Hg)	74.8 ± 7.9*	80.3 ± 9.6*	1.82	.071
Heart rate baseline (bpm)	88.4 ± 6.8	88.7 ± 7.3	0.53	.601
Heart rate posttreatment (bpm)	82.1 ± 5.7*	84.9 ± 6.6*	2.04	**.046**

* The difference after treatment was statistically significant compared with baseline data (*P* < .05).

### 3.3. Comparison of pain and anxiety scores before and after treatment in the 2 groups

At baseline, there were no significant differences in pain and anxiety scores between the experimental and control groups. The baseline pain score was 5.0 ± 1.2 in the experimental group and 5.1 ± 1.3 in the control group (*P* = .730). Similarly, the baseline anxiety score was 4.8 ± 1.1 in the experimental group and 4.9 ± 1.0 in the control group (*P* = .680), indicating comparable initial conditions.

After treatment, significant differences emerged. The pain score in the experimental group decreased to 3.2 ± 1.1, significantly lower than the posttreatment pain score in the control group, which was 4.5 ± 1.3 (*P* = .012). The anxiety score in the experimental group decreased to 2.8 ± 0.9, also significantly lower than the posttreatment anxiety score in the control group, which was 3.6 ± 1.0 (*P* = .021). These results indicate that systematic nursing intervention effectively reduced patients’ pain and anxiety compared to standard care (Table 3).

**Table 3:** Baseline and posttreatment comparison of pain and anxiety scores between experimental and control groups.

Variable	Experimental group (n = 34)	Control group (n = 34)	*t*-value	*P*-value
Pain score baseline (mean ± SD)	5.0 ± 1.2	5.1 ± 1.3	0.35	.73
Pain score posttreatment (mean ± SD)	3.2 ± 1.1*	4.5 ± 1.3*	2.65	.012
Anxiety score baseline (mean ± SD)	4.8 ± 1.1	4.9 ± 1.0	0.41	.68
Anxiety score posttreatment (mean ± SD)	2.8 ± 0.9*	3.6 ± 1.0*	2.34	.021

* The difference after treatment was statistically significant compared with baseline data (*P* < .05).

### 3.4. Comparison of serum amylase, CRP, and white blood cell levels before and after treatment in the 2 groups

At baseline, there were no significant differences in serum amylase, CRP, and white blood cell (WBC) levels between the experimental and control groups. The baseline serum amylase level in the experimental group was 180.2 ± 45.1, while in the control group it was 182.5 ± 46.3 (*P* = .810). Similarly, the baseline CRP level was 15.8 ± 4.5 in the experimental group and 16.1 ± 4.8 in the control group (*P* = .795), and the baseline WBC levels were 12.3 ± 2.8 and 12.7 ± 3.0, respectively (*P* = .570).

After treatment, the experimental group showed significant reductions. The serum amylase level in the experimental group decreased to 95.4 ± 30.2, significantly lower than the posttreatment level of 120.5 ± 35.8 in the control group (*P* = .004). Similarly, the CRP level in the experimental group decreased to 7.2 ± 3.1, while in the control group it was 10.3 ± 3.5 (*P* = .005). The WBC count in the experimental group decreased to 8.5 ± 2.0, while the control group’s WBC count remained higher at 10.1 ± 2.6, with a statistically significant difference (*P* = .007) (Table 4).

**Table 4:** Baseline and posttreatment comparison of serum amylase, CRP, and white blood cell levels between experimental and control groups.

Variable	Experimental group (n = 34)	Control group (n = 34)	Z-value	*P*-value
Serum amylase baseline (mean ± SD)	180.2 ± 45.1	182.5 ± 46.3	0.24	.81
Serum amylase posttreatment (mean ± SD)	95.4 ± 30.2*	120.5 ± 35.8*	3.12	**.004**
CRP baseline (mean ± SD)	15.8 ± 4.5	16.1 ± 4.8	0.26	.795
CRP posttreatment (mean ± SD)	7.2 ± 3.1*	10.3 ± 3.5*	3.04	**.005**
White blood cells baseline (mean ± SD)	12.3 ± 2.8	12.7 ± 3.0	0.57	.57
White blood cells posttreatment (mean ± SD)	8.5 ± 2.0*	10.1 ± 2.6*	2.85	**.007**

* The difference after treatment was statistically significant compared with baseline data (*P* < .05).

### 3.5. Complication rates in the experimental and control groups

Patients in the experimental group who received systematic nursing care had a generally lower incidence of complications than those in the control group. The incidence of MODS complications was 23.53% in the experimental group, significantly lower than the 73.53% in the control group (*P* = .030). The infection rate in the experimental group was 29.41%, compared to 88.24% in the control group (*P* = .014). A similar trend was observed for renal insufficiency, with an incidence of 14.71% in the experimental group and 58.82% in the control group (*P* = .026). The bleeding rate was also significantly lower in the experimental group at 8.82%, compared to 44.12% in the control group (*P* = .040). For hypotension, the incidence in the experimental group (14.71%) was lower than in the control group (52.94%), though the difference was close to but did not reach statistical significance (*P* = .058). These findings suggest that systematic nursing can effectively reduce certain complications in SAP patients (Table 5).

**Table 5:** Comparison of complication incidence rates between experimental and control groups.

Complication type	Cases in experimental group	Incidence rate in experimental group (%)	Cases in control group	Incidence rate in control group (%)	Chi-square value	*P*-value
MODS	8	23.53	25	73.53	4.73	**.03**
Infection	10	29.41	30	88.24	6	**.014**
Renal dysfunction	5	14.71	20	58.82	4.97	**.026**
Bleeding	3	8.82	15	44.12	4.22	**.04**
Hypotension	5	14.71	18	52.94	3.61	.058
Hypothermia	4	11.76	14	41.18	2.32	.127
Catheter blockage	2	5.88	10	29.41	2.29	.13
Catheter-related Bloodstream infection	3	8.82	12	35.29	2.28	.131
Electrolyte imbalance	4	11.76	15	44.12	2.91	.088
Excessive circulatory load	2	5.88	9	26.47	1.71	.192

MODS = multiple organ dysfunction syndrome.

### 3.6. Comparison of nursing satisfaction between the experimental and control groups

**Figure 2.**
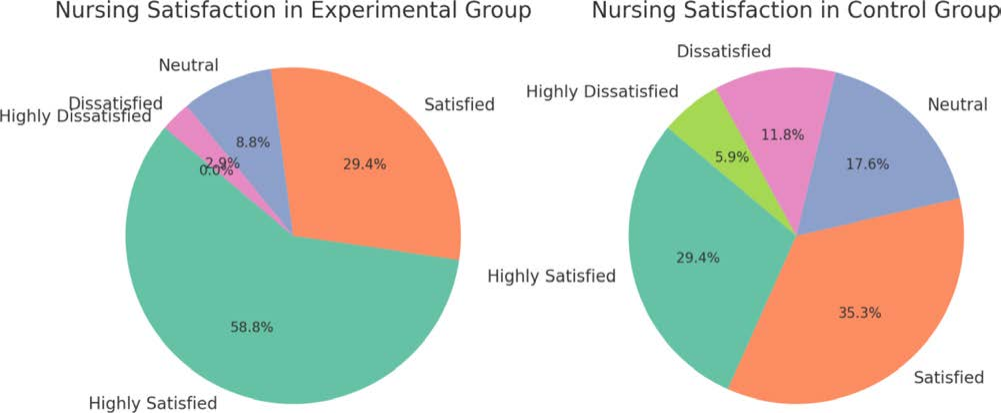
Comparison of nursing satisfaction between the 2 groups.

There was a significant difference in nursing satisfaction between the experimental and control groups. In the experimental group, 58.8% of patients were classified as highly satisfied, compared to only 29.4% in the control group. Satisfaction was reported by 29.4% of patients in the experimental group, slightly lower than 35.3% in the control group. For neutral satisfaction, 8.8% of patients in the experimental group reported this level, compared to 17.6% in the control group. The percentages of dissatisfied (2.9%) and highly dissatisfied (0.0%) patients were lower in the experimental group, while in the control group, the dissatisfied and highly dissatisfied rates were 11.8% and 5.9%, respectively. Chi-square analysis indicated a trend of improved satisfaction in the experimental group (chi-square = 8.32), with a statistically significant difference (*P* = .041) (Table 6) (Fig. 2).

**Table 6:** Comparison of nursing satisfaction levels between experimental and control groups.

Satisfaction level	Experimental group (n = 34)	Control group (n = 34)	Percentage in experimental group (%)	Percentage in control group (%)	Chi-square value	*P*-value
Highly satisfied	20	10	58.8	29.4		
Satisfied	10	12	29.4	35.3		
Neutral	3	6	8.8	17.6		
Dissatisfied	1	4	2.9	11.8		
Highly dissatisfied	0	2	0	5.9	8.32	**.041**

## 4. Discussion

SAP is a common acute abdominal disease with high mortality and complication rates. Its pathological mechanism is complex, often involving SIRS and MODS.^[15]^ Blood purification therapy is a commonly used treatment for SAP patients, aiding in the removal of metabolic waste, controlling inflammation, and maintaining fluid balance. However, the quality of nursing interventions during blood purification treatment plays a crucial role in the effectiveness of the therapy.^[16]^

The results of this study demonstrate the significant benefits of systematic nursing for SAP patients undergoing blood purification. By comparing outcomes between patients receiving systematic nursing (experimental group) and those receiving routine nursing (control group), we found that systematic nursing intervention improved multiple aspects of patient outcomes, including stability of vital signs, reduction in pain and anxiety, control of the inflammatory response, incidence of complications, and nursing satisfaction.

Firstly, the stability of vital signs, especially temperature and heart rate, was significantly better in the systematic nursing group than in the control group. This enhanced stability can be attributed to comprehensive monitoring and proactive adjustments by nursing staff, ensuring adequate management of patients during CRRT. By maintaining a stable temperature and controlling heart rate, systematic nursing helps improve hemodynamic stability in SAP patients, which is crucial for preventing complications like SIRS, which can lead to hemodynamic instability.

Secondly, systematic nursing significantly reduced the levels of pain and anxiety in SAP patients. Comprehensive interventions, including detailed patient education, psychological support, and active pain management strategies, contributed to alleviating patient discomfort. Reducing pain and anxiety not only improved patient experience and quality of life but may also contribute to better clinical outcomes, as lower stress levels are often associated with stronger immune function and a more effective recovery process. The significant improvement in anxiety scores also underscores the importance of emotional support and patient-centered communication, which are key components of the systematic nursing approach.

Compared to the control group, the experimental group showed a significant reduction in inflammatory markers (such as serum amylase, C-reactive protein [CRP], and white blood cell count) posttreatment. These findings indicate that systematic nursing interventions, including strict infection control measures and precise fluid management, are effective in controlling inflammation and reducing the systemic inflammatory burden.^[17–19]^ By more effectively managing inflammation, systematic nursing may help mitigate the risk of common and serious complications in SAP patients, such as MODS.

The incidence of complications (including MODS, infections, renal insufficiency, and bleeding) in the experimental group was significantly lower than in the control group. This suggests that systematic nursing interventions play a crucial role in preventing complications associated with CRRT in SAP patients. The comprehensive approach of systematic nursing, which includes proactive monitoring, rapid response to potential complications, and individualized care planning, likely contributes to these results. For example, frequent assessment and management of catheter-related issues help minimize the risk of catheter blockages and infections, which are common complications in CRRT.^[20–23]^

Patient satisfaction with nursing care was significantly higher in the experimental group than in the control group. This increased satisfaction may be due to personalized care, continuous communication, and timely responsiveness to patient needs provided by the systematic nursing team. Higher satisfaction reflects not only an improved patient experience but is also associated with better adherence to treatment protocols, thereby enhancing overall clinical outcomes. Patients who feel well-cared-for are more likely to trust their healthcare providers, cooperate with treatment, and report better overall health outcomes.

### 4.1. Limitations of the study

This study has several limitations. First, the retrospective design may introduce selection bias; although PSM was used to minimize baseline differences, unmeasured confounding factors could still impact the results. Second, the sample size is relatively small, and the study was conducted at a single center, which may limit the generalizability of the findings. Future studies should consider larger, multicenter prospective trials to validate these results and explore the long-term effects of systematic nursing on patient outcomes. Additionally, integrating patient-reported outcomes and quality-of-life measures with longer follow-up periods could provide more comprehensive insights into the benefits of systematic nursing for SAP patients. This study primarily focused on the short-term effects of systematic nursing interventions, which limits our understanding of their sustained impact on patient rehabilitation. Long-term follow-up studies are needed to evaluate outcomes such as recurrence rates, quality of life, and functional recovery over extended periods. For example, assessing whether the benefits observed in the acute phase translate into long-term improvements in patient well-being would provide a more comprehensive understanding of the intervention’s efficacy. However, due to constraints in time and resources, our study did not include long-term follow-up, which is a notable limitation. Future research should prioritize longitudinal studies to explore the durability of nursing interventions and their potential to reduce long-term healthcare burdens.

Although our study has demonstrated the efficacy of systematic nursing interventions in improving patient outcomes, the lack of cost-effectiveness analysis remains a limitation. Future studies should incorporate a comprehensive evaluation of resource consumption and cost-effectiveness to better inform clinical decision-making and policy development. For instance, comparing the costs associated with different intervention models (e.g., standard care vs systematic nursing) could provide valuable insights into the economic feasibility of implementing such programs in diverse healthcare settings. Additionally, cost-effectiveness analyses could help identify the most efficient allocation of resources, ensuring that the benefits of systematic nursing interventions are maximized while minimizing financial burdens on healthcare systems. By addressing these economic aspects, the widespread adoption of systematic nursing interventions could be further facilitated, particularly in resource-limited settings.

## 5. Conclusion

In summary, systematic nursing significantly improved the clinical outcomes of SAP patients undergoing CRRT by enhancing the stability of vital signs, reducing pain and anxiety, controlling inflammation, lowering complication rates, and increasing nursing satisfaction. These findings highlight the importance of adopting systematic and individualized nursing approaches in the management of critically ill patients.

## Author contributions

**Conceptualization:** Lan Fang, Mengmeng Wang, Xiaoling Ye, Liang He.

**Data curation:** Lan Fang, Mengmeng Wang, Liang He.

**Formal analysis:** Lan Fang, Mengmeng Wang, Liang He.

**Funding acquisition:** Xiaoling Ye, Liang He.

**Investigation:** Lan Fang, Mengmeng Wang, Xiaoling Ye, Jiehua Wu.

**Methodology:** Lan Fang, Xiaoling Ye.

**Supervision:** Xiaoling Ye, Jiehua Wu, Liang He.

**Validation:** Xiaoling Ye, Jiehua Wu, Liang He.

**Writing – original draft:** Lan Fang, Liang He.

**Writing – review & editing:** Lan Fang, Liang He.
